# Recognizing clinical entities in hospital discharge summaries using Structural Support Vector Machines with word representation features

**DOI:** 10.1186/1472-6947-13-S1-S1

**Published:** 2013-04-05

**Authors:** Buzhou Tang, Hongxin Cao, Yonghui Wu, Min Jiang, Hua Xu

**Affiliations:** 1 School of Biomedical Informatics, The University of Texas Health Science Center at Houston, Houston, Texas, USA; 2 Harbin Institute of Technology Shenzhen Graduate School, Shenzhen, China; 3 Second Military Medical University Shanghai, China

## Abstract

**Background:**

Named entity recognition (NER) is an important task in clinical natural language processing (NLP) research. Machine learning (ML) based NER methods have shown good performance in recognizing entities in clinical text. Algorithms and features are two important factors that largely affect the performance of ML-based NER systems. Conditional Random Fields (CRFs), a sequential labelling algorithm, and Support Vector Machines (SVMs), which is based on large margin theory, are two typical machine learning algorithms that have been widely applied to clinical NER tasks. For features, syntactic and semantic information of context words has often been used in clinical NER systems. However, Structural Support Vector Machines (SSVMs), an algorithm that combines the advantages of both CRFs and SVMs, and word representation features, which contain word-level back-off information over large unlabelled corpus by unsupervised algorithms, have not been extensively investigated for clinical text processing. Therefore, the primary goal of this study is to evaluate the use of SSVMs and word representation features in clinical NER tasks.

**Methods:**

In this study, we developed SSVMs-based NER systems to recognize clinical entities in hospital discharge summaries, using the data set from the concept extration task in the 2010 i2b2 NLP challenge. We compared the performance of CRFs and SSVMs-based NER classifiers with the same feature sets. Furthermore, we extracted two different types of word representation features (clustering-based representation features and distributional representation features) and integrated them with the SSVMs-based clinical NER system. We then reported the performance of SSVM-based NER systems with different types of word representation features.

**Results and discussion:**

Using the same training (N = 27,837) and test (N = 45,009) sets in the challenge, our evaluation showed that the SSVMs-based NER systems achieved better performance than the CRFs-based systems for clinical entity recognition, when same features were used. Both types of word representation features (clustering-based and distributional representations) improved the performance of ML-based NER systems. By combining two different types of word representation features together with SSVMs, our system achieved a highest F-measure of 85.82%, which outperformed the best system reported in the challenge by 0.6%. Our results show that SSVMs is a great potential algorithm for clinical NLP research, and both types of unsupervised word representation features are beneficial to clinical NER tasks.

## Background

Recently, rapid growth of large electronic health records (EHRs) has led to an unprecedented expansion of the availability of electronic medical data, including clinical narratives. EHR data have been used not only to support computerized clinical applications (e.g., clinical decision support systems), but also to enable clinical and translational research. One of the challenges for using EHR data is that much of detailed patient information is embedded in clinical text, which is not directly accessible for other computerized applications that reply on structured data. Therefore, natural language processing (NLP) technologies, which can extract structured clinical information from narrative text, have been introduced to the medical domain for more than a decade [1]. Many clinical NLP systems have been developed and used in different applications [2].

Named Entity Recognition (NER), which is to identify boundary and to determine semantic classes (e.g., person names, locations, or organizations) of words/phrases in free text, is an important task in NLP research. Apparently, recognition of clinical entities such as drugs and diseases in clinical text is one of the fundamental tasks for clinical NLP systems as well. Most existing clinical NLP systems (e.g., MedLEE [1], SymText/MPlus [3,4], MetaMap [5] and KnowledgeMap [6]), as well as recent open source ones such as cTAKES [7] and HiTEX [8] often use rule-based methods that rely on existing biomedical vocabularies to identify clinical entities. More recently, i2b2 (the Center of Informatics for Integrating Biology and the Bedside) at Partners Health Care System has organized a few clinical NLP challenges that aimed to recognize clinical entities from text, including the 2009 challenge on medication recognition [9] and the 2010 i2b2 challenge on recognizing medical problems, treatments, and tests entities [10]. In the 2009 challenge, both rule-based [11,12] and machine learning based methods [13,14], as well as hybrid methods [15] have been developed to extract medication entities. In the 2010 i2b2 NLP challenge, organizers provided more annotated data. Therefore, many participating teams, including all top five systems (with F-measures ranging from 81.3% to 85.2%), were primarily based on machine learning approaches [16-18].

To apply machine learning algorithms to an NER task, annotated data are typically converted into a BIO format. Specifically, it assigns each word into a class as follows: **B **means beginning of an entity, **I **means inside an entity, and **O **means outside of an entity. By doing that, an NER problem now can be considered as a classification problem of sequential labeling, which assigns one of the three class labels to each word. Different machine learning algorithms have been used for NER tasks. Among them, Conditional Random Fields (CRFs) and Support Vector Machines (SVMs) are two widely used algorithms. In NER tasks for biomedical literature corpus, some studies reported better results using CRFs [19], while others showed that the SVMs was better [20]. In theory, CRFs is a representative sequence labeling algorithm, which is suitable for the NER problem. SVMs is a robust machine learning algorithm that is designed for classification tasks based on large margin theory. By default, it ignores the relationships between neighbor tokens in sequences when we apply it to sequence labeling problems, although researchers have developed methods to incorporate neighbour information into features for SVMs-based NER systems [21,22]. In 2005, Structural Support Vector Machines (SSVMs) was proposed by Tsochantaridis et al. [23] for structural data, such as trees and sequences. It is an SVMs-based discriminative algorithm for structural prediction. Therefore, SSVMs combines the advantages of both CRFs and SVMs and is suitable for sequence labeling problems. Recently, SSVMs has been applied to NER tasks in different domains and sometimes it shows improved performance when it is compared with CRFs [23]. However, the use of SSVMs for clinical entity recognition has not been extensively evaluated yet.

Another important factor that largely affects the performance of ML-based NER systems is features used to train the model. Syntactic (e.g., part-of-speech tags) and semantic (e.g., semantic classes in UMLS (Unified Medical Language System)) information of context words are often used as features in clinical NER systems. However, word representation, which generates word-level back-off features over large unlabeled corpus by unsupervised algorithms, has not been widely used. This type of features often contains grammatical or semantic meanings, and can represent words that do not appear in the labelled corpus effectively. Different techniques have been used to generate word representation features. For example, Joseph et al. [24] classified them into three categories: clustering-based, distributional and distributed word representations. Word representation features have been used in NLP work in the general English domain, and have shown stable improvements on a variety of tasks [25,26]. However, few studies have applied word representation features to NLP research in the medical domain. de Bruijn B et al. [16] used some clustering-based word representation features in their NER system for the 2010 i2b2 NLP challenge and achieved the highest performance in the challenge. Jonnalagadda et al. [27] investigated distributional semantics features for clinic entity recognition, and their evaluation on the same 2010 i2b2 challenge data showed a significant improvement when using these features. Nevertheless, the contribution of different types of word representation features to clinic entity recognition has not been extensively investigated yet.

In our previous work presented in the ACM sixth international workshop on Data and text mining in biomedical informatics (DTMBIO'12) [28], we explored the uses of SSVMs, combined features, clustering-based word representation features and tag representations for clinical entity recognition. This paper is an extension to our previous work [28]. In addition to the comparison between SSVMs and CRFs, we implemented two types of word representation features (clustering-based and distributional word representation features) and evaluated the contribution of individual and combined word representation features from these two different methods, for clinic entity recognition. Our results showed that SSVMs achieved higher performance than CRFs on the 2010 i2b2 concept extraction data set, indicating it is a promising alternative algorithm for clinical entity recognition. In addition, we demonstrated not only that both clustering-based and distributional word representation features were beneficial to clinical NER tasks, but also that these two types of word representation features were complementary to each other. When both types of word representation features were combined with SSVMs, our system achieved a highest F-measure of 85.82%, an improvement of 0.4% to the baseline system, which outperformed the best system reported in the challenge by 0.6%.

## Methods

### Data sets

In this study, we applied SSVMs to the Concept Extraction task of the 2010 i2b2 NLP challenge. The task was to extract clinical entities including Problem, Test, and Treatment from clinical narratives, which included discharge summaries and some progress notes obtained from three institutions: Partners HealthCare, Beth Israel Deaconess Medical Center, and University of Pittsburgh Medical Center. We used the same training and test data sets as in the challenge, which consisted of 349 notes for training and 477 notes for testing. For each note, annotators manually extracted entities about Problem, Treatment, and Test. Table 1 shows the counts of different types of entities in both training and test data sets.

**Table 1:** Counts of different types of entities in training and test data sets used in this study.

	Concepts (N = 72,846)			
**Data Set**	**Problem**	**Treat**	**Test**	**All**

Training (349 notes)	11,968	8,500	7,369	27,837
Test (477 notes)	18,550	13,560	12,899	45,009

### Machine learning algorithms: SSVMs and CRFs

The task of sequence labeling for NER is to find the best label sequence y* = *y_1_y_2 _... y_N _*for a given input sequence *x = x_1_x_2 _... x_N _*and a set of labels ***L***, where *y_i_*∈***L ***for *1≤i≤N*. In CRFs, y* is the tag sequence with the highest probability for the given input sequence. For SSVMs, y* is the tag sequence with the highest score determined by a linear discriminant function. Both of them decode the sequence-labeling problem by undirected Markov chain and Viterbi algorithm. The difference between them is the optimization goal. SSVMs models sequence labeling problems by the large margin method like SVMs, which has good generalization ability; while CRFs models sequence labeling problems by maximum likelihood estimation of conditional probability, which could suffer from the over-fitting problem.

Taken the first order markov linear-chain for example, the discriminant function of SSVMs is shown below:

(1) $$\begin{array}{c} h_{w}\left(x,y\right)=w^{T}F\left(x,y\right)=\underset{i}{\sum }w_{i}^{T}F\left(x,y_{i}\right)\\ =\underset{i}{\sum }w_{i}^{T}\left(f\left(x_{i},y_{i}\right),f\left(x,y_{i-1}y_{i}\right)\right) \end{array}$$

where $f\left(x,y_{j}\right)$ and $f\left(x,y_{j-1}y_{j}\right)$ are features of a node clique (a fully connected sub-graph) and a pairwise clique in the linear markov chain respectively. Under the fundamental theorem of SVMs, the sequential labeling problem can be formalized as the following optimization problem: given a training dataset of *m *independent identically distributed samples *S*, drawn from a fixed distribution D*_X×Y_*. The optimization goal is:

(2) $$\begin{array}{l} \underset{w,\varepsilon }{\min}\frac{1}{2}{\left\|w\right\|}^{2}+\frac{C}{m}\sum_{x\in S}\xi_{x}\\ s.t.\;w^{T}F(x,y)\geq w^{T}F(x,y^{'})+l(y,y^{'})-\xi_{x},\;\\ \;\forall {\text{y}}^{\text{'}}\neq y,\;\xi_{x}\geq 0\text{, (}x,y)\in S \end{array}$$

where *C *trades off margin size and training error, $l\left(y,y^{\prime }\right)$ is a loss function that computes the distance between *y *and *y'*, and $\xi_{x}$ is a slack variant for non-separable data.

We used SVM*^hmm ^*(http://osmot.cs.cornell.edu/svm_hmm/) as the implementation of SSVMs in this study, which solves Eq.2 by the Cutting-Plane algorithm [23,29]. For CRFs, we used the CRF++ package (http://crfpp.sourceforge.net/), which has been widely used for various NER tasks [30,31]. The pair-wise (one-against-one) strategy for multi-classification was used in this study.

### Tags for entities

**Figure 1:**
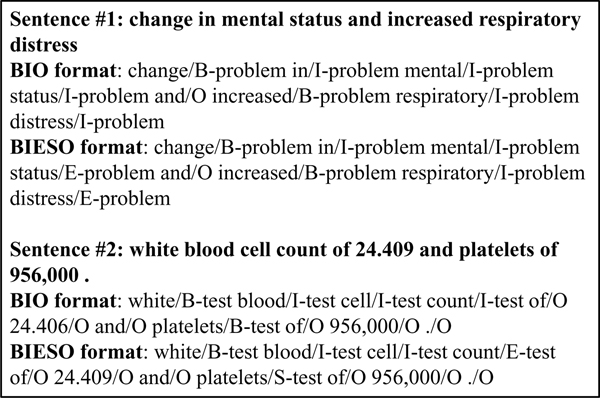
**Examples of two different tag representations: BIO vs. BIESO**.

To convert an NER task to a classification problem, we need to assign annotated entities with appropriate tag representations. The "BIO" format [32] is a commonly used representation for entity tags, in which each word is assigned into a label as following: **B **= beginning of an entity, **I **= inside an entity, and **O **= outside of an entity. As there were three types of entities in our task, we defined three different B classes and three different I classes, resulting in total seven different tags (B-problem, B-test, B-treatment, I-problem, I-test, I-treatment, O). Besides the BIO style, there is another type of tag representation called BIESO (**B**-beginning, **I**-intermediate, **E**-end, **S**-single word entity and **O**-outside), which introduces two additional tags to distinguish "end" tokens from "intermediate" tokens and single word entities from multi-word entities. For the "BIESO" format, it contained 13 different tags (B-problem, I-problem, E-problem, S-problem, B-treatment, I-treatment, E-treatment, S-treatment, B-test, I-test, E-test, S-test, O). Figure 1 shows examples of both BIO and BIESO tag representations for two sentences, where bold words are annotated clinic entities. Although BIO is commonly used in NER tasks, our previous study [28], showed that BIESO had better performance in clinical entity recognition. Therefore, we included both tag representations in this study.

### Features for machine learning classifier

We have developed a CRFs-based classifier for the 2010 i2b2 challenge and it ranked second among 20 participating teams [17]. In that study, we used four types of features including 1) *word level information *such as bag-of-word and orthographic information; 2) *syntactic information *such as Part of Speech (POS) tags; 3) *lexical and semantic information *from NLP systems such as normalized concepts (e.g., UMLS concept unique identifiers (CUIs)) and semantic types; and 4) *discourse information *such as sections in the clinical notes. In [28], we introduced combined features, which were generated by combining different types of features (e.g., word and POS), and it improved performance. Therefore, in this study, our baseline method included all four types of features in [17] and the combined features in [28]. The focus of this study was then to compare the contribution of two types of word representation features: clustering-based and distributional representations.

#### 1) Clustering-based word representation

**Figure 2:**
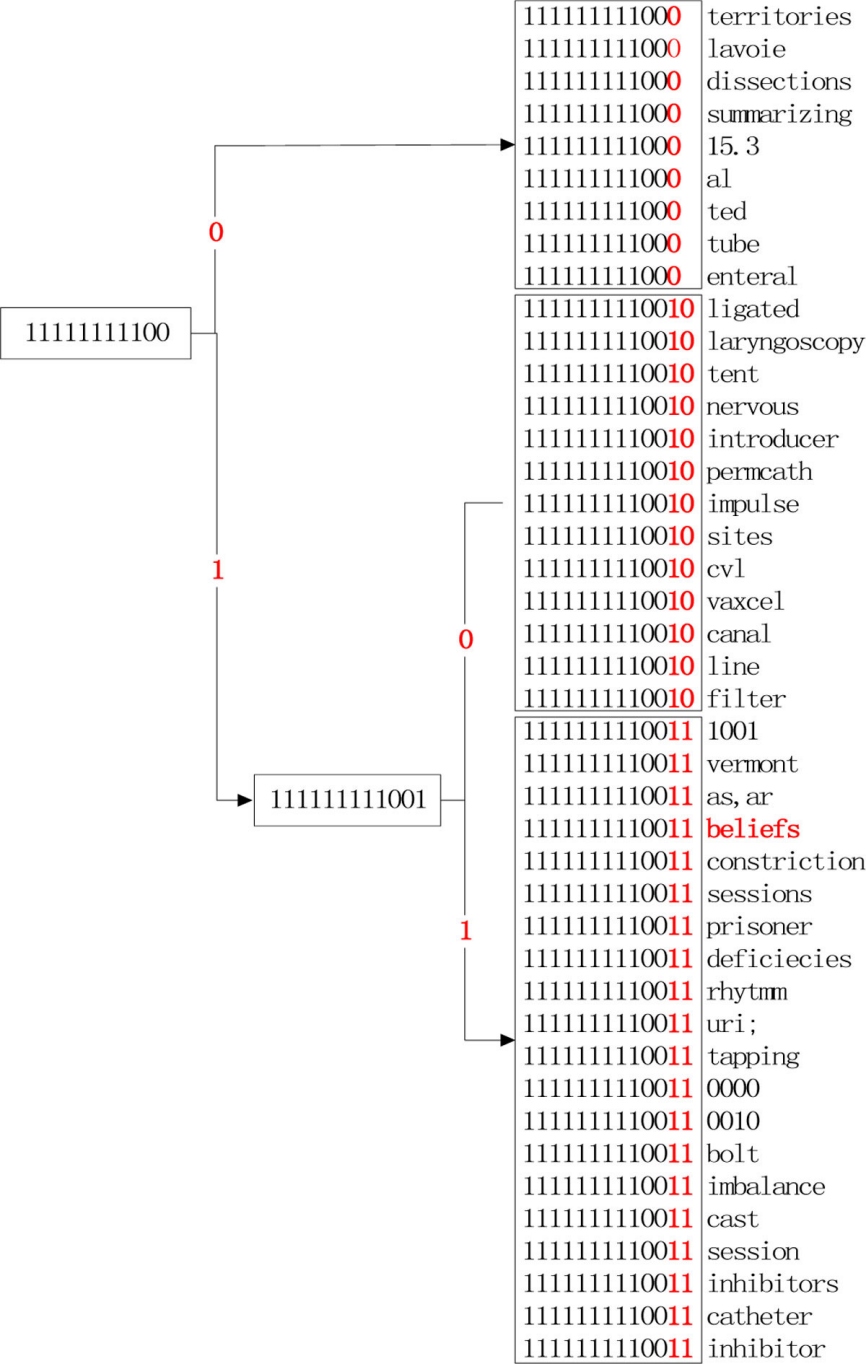
**A hierarchical structure fragment of 42 words**.

This approach is to induce clusters over words in the unlabelled corpus. After that, a word can be represented by the cluster or clusters it belongs to. Similar to [16], we implemented the Brown clustering algorithm [33], a hierarchical clustering algorithm, to generate unsupervised word representation features for the NER systems in this study. After running the Brown clustering algorithm against the corpus, we generated a hierarchical cluster of all the words in the corpus, represented as a binary tree, where all the words are the leaf nodes. Figure 2 shows a hierarchical cluster containing 42 words, which were clustered into 3 clusters. The numbers in the figure (e.g., 111111111000) represent the sub-paths starting from the root of the cluster, which contain valuable information. Words that share similar sub-paths are semantically closer. In our experiments, all sub-paths were used as features to represent each word. For example, the following features were extracted for the word "beliefs": {"1", "11", "111", "1111", "11111", "111111", "1111111", "11111111", "111111111", "1111111110", "11111111100", '111111111001", "1111111110011"}. Different numbers of clusters {50, 100, 200, 500, 1000, 2000} were tested and 1000 was selected as the optimized number of clusters.

#### 2) Distributional word representation

**Figure 3:**
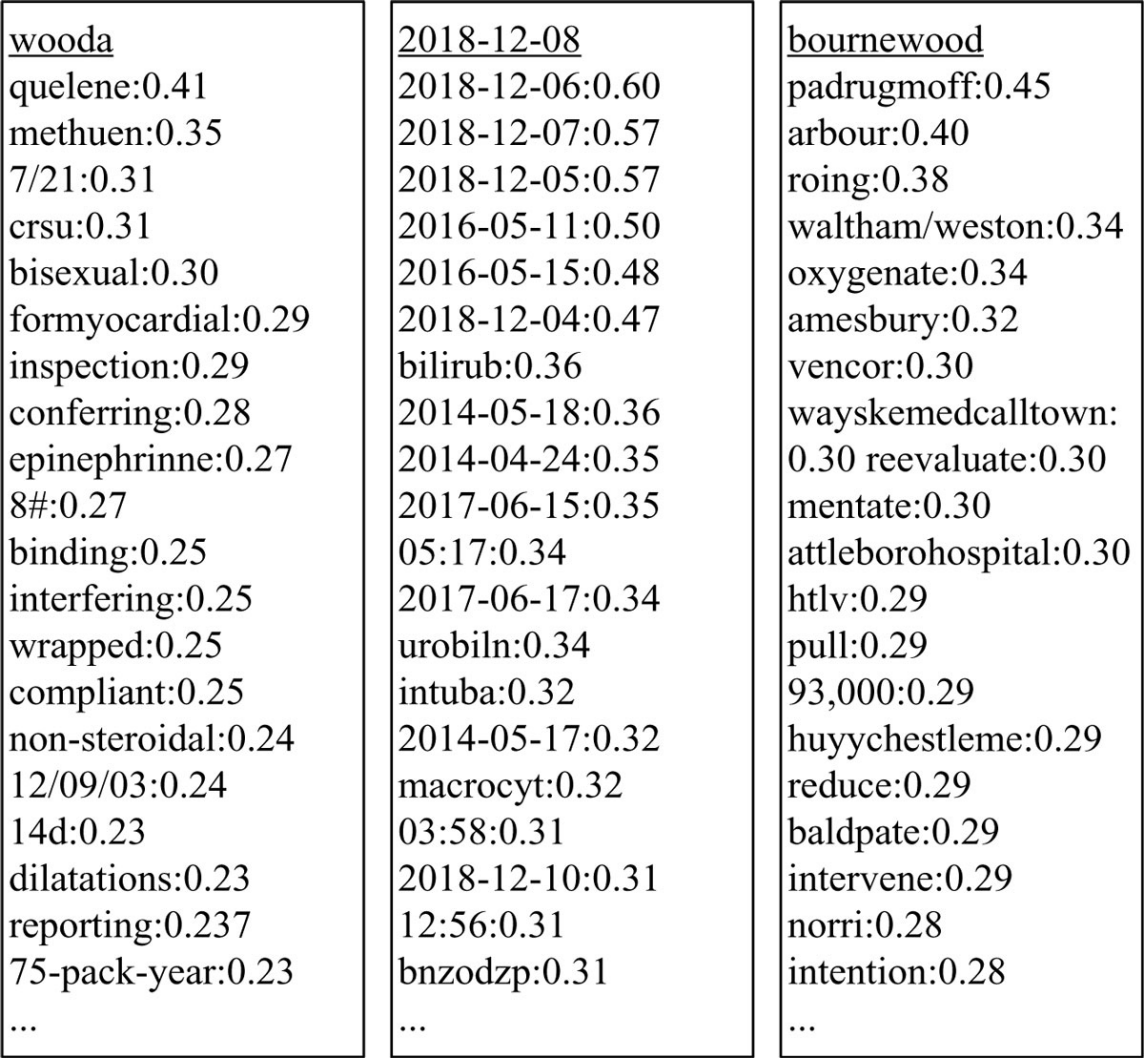
**A fragment of the semantic thesaurus of three words**.

This is a word co-occurrence based approach to latent semantics. Statistical approximations are used to reduce a word co-occurrence matrix of high dimensionality to a latent semantic matrix of low dimensionality. After the reduction, semantic thesaurus from the semantic matrix can be constructed by computing similarities of each two words, or clusters can be generated on the semantic matrix, similar to the clustering-based word representation. A word then can be represented by other words in the semantic thesaurus or cluster(s). In this study, we represented a word by its nearest semantic words in the semantic thesaurus. Random indexing was used for reduction, and cosine function was used for semantic similarities. Figure 3 shows a fragment of the semantic thesaurus of three words from the 2010 i2b2 NLP challenge corpus. The words with underline (e.g. wooda) are words appearing in the corpus, and other words underneath (e.g. quelene) are words in the semantic thesaurus, sorted by semantic similarity (e.g. 0.41). In our experiments, we used the same optimized parameters as [34], which also ran random indexing on the same corpus. Each word was represented by its 20 nearest semantic words in the thesaurus. For example, the following feature were extracted for the word "wooda": {"quelene", "methuen", "7/21", "crsu", "bisexual", "formyocardial", "inspection", "conferring", "epinephrinne", "8#", "binding", "interfering", "wrapped", "compliant", "non-steroidal", "12/09/03", "14d", "dilatations", "reporting", "75-pack-year"}.

### Experiments and evaluation

For each algorithm (SSVMs vs. CRFs), we developed the NER system using the training data set containing 349 annotated clinical notes and evaluated it using the test set of 477 annotated notes. Same feature files were used for both algorithms. Parameters for each algorithm were optimized using the training set via a 10-fold cross validation (CV) method. To evaluate the effect of different types of word representation features, we started with the baseline method that used four types of features and the combined features described in the previous section, and then added two types of unsupervised word representation features and reported results correspondingly. We also compared performance of different algorithms when either BIO or BIESO tags were used. All experiments were conducted on computers of Intel(R) Xeon(R) CPU X5670 @ 2.93 GHz.

Micro-averaged Precision, Recall, and F-measure for all concepts were reported by using the evaluation script provided by i2b2 challenge organizers [9]. Results were reported using both exact matching, which requires that the starting and ending offsets of a concept have to be exactly same as those in the gold standard, and inexact matching, which refers to cases where their offsets are not exactly same as those in gold standard, but they overlap with each other. To assess whether the mean F-measures of both NER systems (using SSVMs or CRFs) are statistically significantly different, we further conducted a statistical test based on results from bootstrapping. From the test set, we randomly selected 2000 sentences with replacement for 200 times and generated 200 bootstrapping data sets. For each bootstrapping data set, we evaluated and measured F-measures for both SSVMs and CRFs based NER systems. We then used Wilcoxon signed rank test [35], a nonparametric test for paired samples, to determine if the difference between F-measures from SSVMs and CRFs based NER systems is statistically significant (p-value < 0.05).

## Results

Table 2 shows the performance of both SSVMs and CRFs based clinical entity recognition systems on the test set, when different features and tag representations were used. The numbers in column 3 and 4 are F-measures followed by corresponding Recall and Precision values in a parenthesis for all concepts, when exact-matching criterion was used. When same features and tags were used, SSVMs consistently showed better F-measures than CRFs. For example, when basic features and BIESO tags were used, SSVMs outperformed CRFs by 0.38% in F-measure. If all features and BIESO tags were used, SSVMs still showed better F-measure than CRFs (85.82% vs. 85.68%), although the difference was smaller. The Wilcoxon signed rank test based on bootstrapping data showed that the improvement of F-measure (SSVMs over CRFs) was statistically significant (p-value < 0.05). For each algorithm, BIESO tags had better performance than that of BIO tags, similar to what we observed in our previous study [28].

**Table 2:** Performance of SSVMs and CRFs based NER systems when different features and tag representations were used.

Tags	Features	SSVMs - F(R/P)(%)	CRFs - F(R/P)(%)
BIO	Base	84.89(83.39/86.44)	84.62 (82.35/87.01)
	Base + Clustering	85.22(84.05/86.43)	85.16 (82.94/87.50)
	Base + Distributional	85.19(84.00/86.42)	85.12(82.80/87.58)
	Base + Clustering + Distributional	**85.45(84.30/86.63)**	**85.31(83.19/87.54)**

BIESO	Base	85.42(83.60/87.31)	85.04(82.31/87.97)
	Base + Clustering	85.74(84.15/87.40)	85.59(83.16/88.16)
	Base + Distributional	85.74(84.16/87.38)	85.35(82.82/88.05)
	Base + Clustering + Distributional	**85.82(84.31/87.38)**	**85.68(83.30/88.20)**

Moreover, both clustering-based and distributional word representation features improved performance of NER systems. In the BIO setting, adding the clustering-based and distributional word representation features improved the performance of SSVMs-based NER systems by 0.33% and 0.30% of F-measure respectively. In the BIESO setting, the improvements were 0.32% for either the clustering-based or distributional word representation features. Moreover, when both types of word representation features were added to the NER systems, the performance improvements were larger than any single type of word representation features, achieving increases of 0.56% and 0.40% F-measure for the BIO and BIESO settings respectively. When all features and BIESO tags were used, both SVMMs and CRFs reached the highest performance. For SSVMs, it achieved a highest exact-matching F-measure of 85.82%, an increase of 0.40% to the baseline method. For CRFs, it achieved that of 85.68%, an increase of 0.64% to the baseline method.

Table 3 shows the detailed results (by entity type) of the best-performed clinical entity recognition systems by either SSVMs or CRFs. These results suggested that SSVMs achieved better F-measures than CRFs across different entity types. However, we also noticed that SSVMs achieved higher recall but lower precision values than CRFs.

**Table 3:** Results by entity type for the best performed SSVMs and CRFs clinical entity recognition systems.

Algorithm	Category	Exact matching (%)			Inexact matching (%)		
		
		Recall	Precision	F-measure	Recall	Precision	F-measure
SSVMs	Overall	84.31	87.38	**85.82**	91.78	93.03	**92.40**
	Problem	86.75	88.50	87.61	93.53	95.29	94.40
	Treatment	85.72	89.27	87.46	91.45	95.17	93.27
	Test	85.13	89.84	87.42	90.26	95.50	92.81

CRFs	Overall	83.30	88.20	**85.68**	90.52	93.96	**92.21**
	Problem	85.73	89.02	87.34	92.46	96.12	94.25
	Treatment	84.14	89.88	86.92	89.99	96.03	92.92
	Test	84.07	90.74	87.28	88.94	95.96	92.32

## Discussion

In this study, we applied SSVMs to clinical entity recognition, and investigated the contribution of two different types of word representation features to this task. Our evaluation using data sets from the 2010 i2b2 NLP challenge shows that SSVMs achieved higher F-measure than CRFs when same features were used, which demonstrated the use of SSVMs for clinical NER tasks. In our study, BIESO tags consistently showed better performance than BIO tags for clinic entity recognition. Either clustering-based or distributional word representation features were of benefit to clinic entity recognition no matter whether SSVMs or CRFs was used. When both of them were added to clinical NER systems, the performance was further improved. When BIESO tags and both word representation features were used, our system achieved the highest F-measure of 85.82%, which is higher than the best system in the 2010 i2b2 challenge [16] by 0.6%. Table 4 shows the comparison between our system and the top five systems in the challenge. We understand that such comparisons may not be fair, as challenge participating teams had limited time to build their systems. But our results suggested that SSVMs and word representation features could be very useful for clinical entity recognition tasks and it is worth investigating its uses for clinical NLP research.

**Table 4:** Comparison between our system and other state-of-the-art systems.

Systems	Algorithm	Exact matching
		
		F-measure (%)
Our system	SSVMs	85.8
deBruijn et al [16]	Semi-Markov	85.2
Jiang et al [17]	CRFs	83.9
Kang et al [36]	CRFs	82.1
Gurulingappa et al [37]	CRFs	81.8
Patrick et al [38]	CRFs	81.3

When comparing SSVMs with CRFs (Tables 2 and 3), we noticed that SSVMs achieved much better recall values, although CRFs usually had better precision values. For sequential labelling problems, SSVMs not only takes advantages of relationships of neighbour words like CRFs, but also has strong generalization ability like SVMs. Different from CRFs, it is not necessary to assume an exponential distribution among training and test data for SSVMs. Therefore, SSVMs has better capability to detecting testing samples that do not appear in the training data. For the clinical NER task in this study, SSVMs found more entities that did not appear in training data than CRFs. For example, when the basic features and clustering-based word representation features were used, SSVMs detected 890 more entities than CRFs. Among them, about 500 entities were true positive. Therefore, SSVMs achieved better recall than CRFs. Given the performance differences in precision and recall of SSVMs and CRFs, they can be complementary to each other. An interesting direction is to combine outputs from SSVMs and CRFs to further improve performance of clinical NER systems, which is one case of our future work.

The performance gain from BIESO tags was not trivial as well (F-measures: 85.42% for BIESO vs. 84.89% for BIO when basic features were used). We noticed that the improvement by BIESO tags was mainly from increased precisions, which indicated that the BIESO tag representation helped the boundary determination of entities. For further details, we looked into all entities (20425 single-word entities and 24584 multi-word entities) in the gold standard. When basic features were used, the precisions of the BIESO-based SSVMs system for sing-word entities and multi-word entities were 91.52% and 87.34% respectively; while the precisions of the BIO-based SSVMs system for single-word and multi-word entities were 90.94%% and 87.33% respectively.

It is not surprising that word representation features such as clustering-based and distributional word representations improved performance of clinical NER systems, as it was reported by previous studies as well [16]. The performance gain from each type of word representation features was not trivial (F-measures: 85.74% for clustering-based or distributional word representation features vs. 85.42% for the the baseline). However, Jonnalagadda et al. [27] reported a larger increase (about 2% F-measure) by using distributional semantics features on the same i2b2 data set. Although the difference in performance gain could be due to different methods for generating word representation features, we would think it is more related to the baseline performance. In Jonnalagadda et al.'s experiment, the baseline method had an F-measure of 80.3%; while our baseline method achieved a much higher F-measure of 85.42%, which made it more difficult for further improvements. We noticed that the improvement by word representation features was mainly from increased recall, which indicated that unsupervised word representation features helped to detect more correct entities; especially those did not appear in the training data set. Moreover, the total performance gain by combining two types of word representation features was a bit higher than the gain from any of them, indicating that these two types of word representation methods could be complementary to each other. To further improve NER performance, it is worth exploring to combine more types of word representation features. In the future, we plan to investigate another type of word representation features: distributed word representation such as Canonical Correlation Analysis (CCA) [36], as well as other algorithms for generating word representations in NLP domain, such as Hyperspace Analogue to Language (HLA) [34].

## Conclusions

In this study, we investigated the use of SSVMs and clustering-based and distributional word representations for clinical entity recognition. Our evaluation using the 2010 i2b2 NLP challenge data showed that SSVMs could achieve better F-measures than CRFs for detecting clinical entities from discharge summaries, indicating its great potential for clinical NLP research. Moreover, we demonstrated not only that both clustering-based and distributional word representation features were beneficial to clinical NER tasks, but also that these two types of word representation features were complementary to each other.

## Competing interests

The authors declare that they have no competing interests.

## Authors' contributions

The work presented here was carried out in collaboration between all authors. BT, HC and HX designed methods and experiments. BT, YW and MJ carried out the experiments. BT, HC and HX analyzed the data, interpreted the results and wrote the paper. All authors have attributed to, seen and approved the manuscript.

## Declarations

The publication costs for this article are funded by the corresponding author.

This article has been published as part of *BMC Medical Informatics and Decision Making *Volume 13 Supplement 1, 2013: Proceedings of the ACM Sixth International Workshop on Data and Text Mining in Biomedical Informatics (DTMBio 2012). The full contents of the supplement are available online at http://www.biomedcentral.com/bmcmedinformdecismak/supplements/13/S1.
